# LPA_3_-mediated lysophosphatidic acid signaling promotes postnatal heart regeneration in mice

**DOI:** 10.7150/thno.47913

**Published:** 2020-08-29

**Authors:** Fang Wang, Si Liu, Jianqiu Pei, Lin Cai, Ning Liu, Tian Liang, Xiaoxuan Dong, Xiangfeng Cong, Jerold Chun, Jinghai Chen, Shengshou Hu, Xi Chen

**Affiliations:** 1 State Key Laboratory of Cardiovascular Disease, Center of Laboratory Medicine, Fuwai Hospital, National Center for Cardiovascular Diseases, Chinese Academy of Medical Sciences and Peking Union Medical College, Beijing 100037, China.; 2 Department of Gastroenterology, Beijing Friendship Hospital, Capital Medical University, National Clinical Research Center for Digestive Disease, Beijing Digestive Disease Center, Beijing Key Laboratory for Precancerous Lesion of Digestive Disease, Beijing, 100050, China.; 3 Sanford Burnham Prebys Medical Discovery Institute, La Jolla, CA, 92037, United States.; 4 Department of Cardiology, The Second Affiliated Hospital, Institute of Translational Medicine, Zhejiang University School of Medicine, 310029 Hangzhou, China.

**Keywords:** Lysophosphatidic acid, LPA receptor, cardiomyocyte, proliferation, heart regeneration

## Abstract

**Background:** Lysophosphatidic acid (LPA) is a small glycerophospholipid that acts as a potent extracellular signal in various biological processes and diseases. Our previous work demonstrated that the expression of the LPA receptors LPA_1_ and LPA_3_ is elevated in the early postnatal heart. However, the role of this stage-specific expression of LPA_1_ and LPA_3_ in the heart is unknown.

**Methods and Results:** By using LPA_3_ and LPA_1_ knockout mice, and neonatal SD rats treated with Ki16425 (LPA_1_/LPA_3_ inhibitor), we found that the number of proliferating cardiomyocytes, detected by coimmunostaining pH3, Ki67 or BrdU with cardiac troponin T, was significantly decreased in the LPA_3_ knockout mice and the Ki16425-treated rats but not in the LPA_1_ knockout mice during the first week of postnatal life. Using a myocardial infarction (MI) model, we found that cardiac function and the number of proliferating cardiomyocytes were decreased in the neonatal LPA_3_ KO mice and increased in the AAV9-mediated cardiac-specific LPA_3_ overexpression mice. By using lineage tracing and AAV9-LPA_3_, we further found that LPA_3_ overexpression in adult mice enhances cardiac function and heart regeneration as assessed by pH3-, Ki67-, and Aurora B-positive cardiomyocytes and clonal cardiomyocytes after MI. Genome-wide transcriptional profiling and additional mechanistic studies showed that LPA induces cardiomyocyte proliferation through the PI3K/AKT, BMP-Smad1/5, Hippo/YAP and MAPK/ERK pathways *in vitro*, whereas only ERK was confirmed to be activated by LPA-LPA_3_ signaling *in vivo*.

**Conclusion:** Our study reports that LPA_3_-mediated LPA signaling is a crucial factor for cardiomyocyte proliferation in the early postnatal heart. Cardiac-specific LPA_3_ overexpression improved cardiac function and promoted cardiac regeneration after myocardial injury induced by MI. This finding suggested that activation of LPA_3_ potentially through AAV-mediated gene therapy might be a therapeutic strategy to improve the outcome after MI.

## Introduction

Lysophosphatidic acid (LPA) is a small glycerolphospholipid that acts as a potent extracellular signaling molecule by binding to the LPA receptor family of G protein-coupled receptors (GPCRs), LPA_1_-LPA_6_ 1. The effects of LPA signaling on cell proliferation, survival, migration, calcium mobilization, and other processes have been described by several studies 2. Expression of LPA_3_ (encoded by the *Lpar3* gene) was observed in the mouse heart by Ohuchi et al 3. LPA signaling was shown to promote the progression of cardiovascular diseases such as hypertension and atherosclerotic plaque formation 4-6. In a previous study, we found that the mRNA and protein levels of LPA_1_ and LPA_3_ peaked during the early postnatal period and decreased rapidly thereafter in the rat heart 7. However, the role of this stage-specific expression of LPA_1_ and LPA_3_ in the heart is still unknown. The present study aimed to address this issue.

It is believed that cardiomyocyte proliferation contributes to mammalian heart growth largely during the embryonic period, and cardiomyocyte enlargement is thought to be responsible for growth after birth. However, accumulating evidence has demonstrated that cardiomyocytes still have proliferative potential even after birth 8-12. Since we found that LPA_1_ and LPA_3_ significantly peaked during the early postnatal period and decreased rapidly thereafter, which coincides with the loss of the heart's regenerative potential, the role of LPA signaling in cardiomyocyte proliferation and heart regeneration after birth was elucidated in this study.

## Results

### LPA_3_-mediated LPA signaling is required for cardiomyocyte proliferation in the early postnatal heart

**Figure 1:**
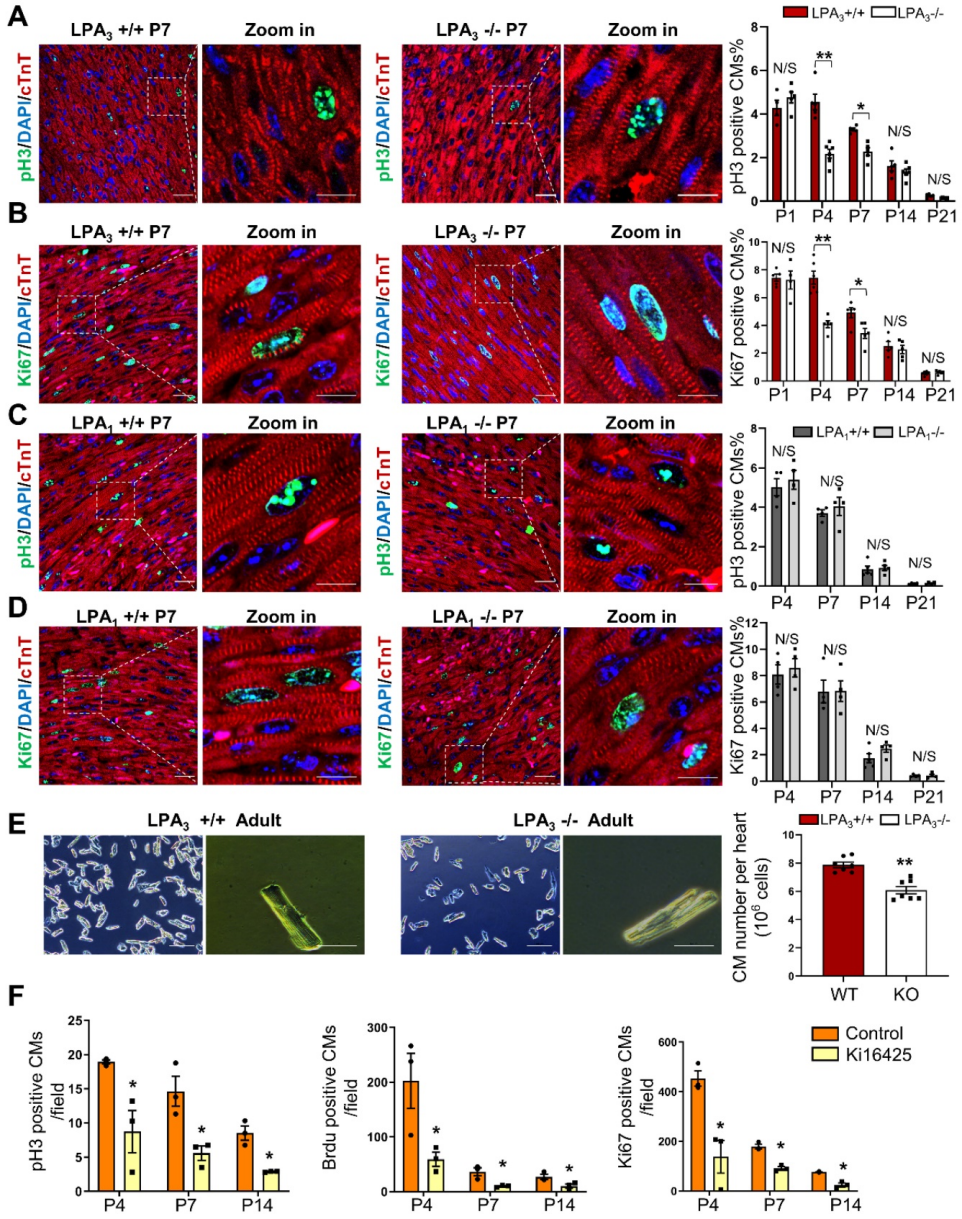
** LPA_3_-mediated LPA signaling is required for cardiomyocyte proliferation in the early postnatal heart. (A, B)** Immunofluorescence and quantification of pH3- and Ki67-positive cardiomyocytes of the LPA_3_ wild-type (WT) and knockout (KO) mice (n = 4-6 per group). **(C, D)** Immunofluorescence and quantification of pH3- and Ki67-positive cardiomyocytes of the LPA_1_ WT and KO mice (n = 4-5 per group). Scale bar of the close-up image =10 µm; scale bar of other images = 20 µm **(E)** The total number of cardiomyocytes of the adult LPA_3_ WT and KO mice (n = 7 per group; scale bar on the left of each group = 200 µm; scale bar on the right of each group = 50 µm). **(F)** Quantification of pH3-, Ki67- and BrdU-positive cardiomyocytes from the P4 to P14 WT and Ki16425-treated rats (n=3 per group). Data are the mean ± SEM; nonsignificant (N/S), *P* > 0.05; “n” stands for the number of mice; each point in the scatter plot indicates the data of individual mice; *P< 0.05; **P<0.01.

We used LPA_3_ and LPA_1_ knockout (KO) mice to explore the potential role of LPA signaling in cardiomyocyte proliferation during postnatal developmental stages of the heart. The proliferation indices of cardiomyocytes from different developmental stages of the heart were examined by colocalization of pH3 and Ki67, which indicate mitosis and cell proliferation, respectively, along with the cardiomyocyte marker cardiac troponin T (cTnT). We found that the number of pH3- (Figure 1A) and Ki67-positive (Figure 1B) cardiomyocytes was significantly decreased in the LPA_3_ KO mice compared to the LPA_3_ wild-type (WT) mice during the first week of postnatal life (52% on P4 and 31% on P7 for pH3 -positive cardiomyocytes; 45% on P4 and 31% on P7 for Ki67-positive cardiomyocytes) but not on day one (P1) or two or three weeks after birth (P14 and P21). In contrast, we did not observe a significant difference in the number of proliferating cardiomyocytes between the LPA_1_ KO mice and the wild-type mice (Figure 1C-D), suggesting that LPA_1_ is unlikely to be directly involved in cardiomyocyte proliferation. Further quantitative analysis demonstrated a significant decrease (31%) in the total number of cardiomyocytes in the adult hearts of the LPA_3_ KO mice compared to the littermate controls (7.87×10^6^ vs 5.41×10^6^, *P* <0.001, Figure 1E). Next, we determined whether loss of LPA_3_ affected the survival of cardiomyocytes. We performed a TUNEL assay to measure apoptosis and observed no change in the TUNEL signals in the P4 and P7 hearts of the LPA_3_ KO mice compared with the controls (Figure S1A).

As a complementary approach, we also examined the role of LPA signaling in cardiomyocyte proliferation by administering Ki16425 (LPA_1_/LPA_3_ inhibitor) to neonatal SD rats. Consistent with the data from the knockout mice, 4 days after Ki16425 intraperitoneal injection at P1, the pH3-, BrdU- and Ki67-positive cardiomyocytes were reduced by 52%, 71% and 70%, respectively (Figure 1F). Together, these data suggest that LPA_3_-mediated LPA signaling is required for cardiomyocyte proliferation in the early postnatal period.

### LPA_3_-mediated cardiomyocyte proliferation is necessary for cardiac regeneration in neonates after myocardial infarction

**Figure 2:**
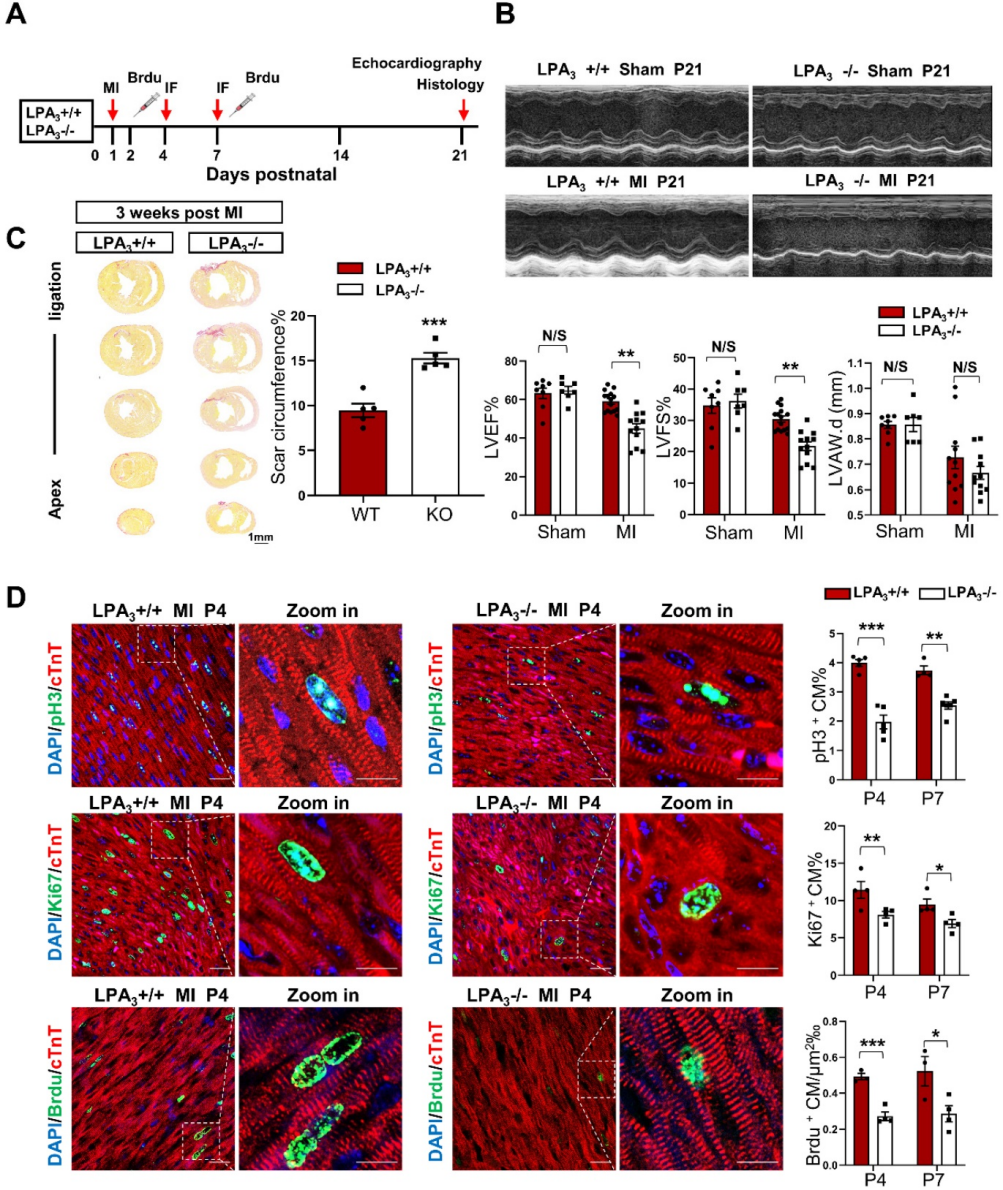
** Cardiac function and cardiomyocyte proliferation decrease in the neonatal LPA_3_ KO mice after myocardial infarction. (A)** Experimental design and timeline of MI in the neonatal mice. MI, myocardial infarction. IF, immunostaining. **(B)** Echocardiography assessment of the cardiac function of the LPA_3_ WT and KO mice 21 days after MI (sham n = 7, MI n = 12-15 per group). LVEF, left ventricular ejection fraction. LVFS, left ventricular fractional shortening. LVPW.d, left ventricular posterior wall diameter in diastole. **(C)** Scale size of the LPA_3_ WT and KO mice at 21 days after MI (n = 5 per group). Scale bar = 1 mm. **(D)** Immunofluorescence and quantification of pH3-, Ki67-, and BrdU-positive cardiomyocytes at P4 and P7 after surgery in the LPA_3_ WT and KO mice (n = 4-6 per group, scale bar of the images = 20 µm; scale bar of the close-up image = 10 µm). Data are the mean ± SEM; nonsignificant (N/S), *P* > 0.05; “n” indicates the number of mice; each point in the scatter plot indicates the data of individual mice; *P< 0.05; **P<0.01; ***P<0.001.

The murine neonatal heart can regenerate lost myocardium and recover cardiac function after injury, whereas this capacity is substantially diminished after the first week 13, 14. Thus, myocardial infarction in neonatal mice has become a classic model for the study of cardiac regeneration. Here, using LPA_3_ knockout mice, we further examined whether LPA_3_-mediated cardiomyocyte proliferation is required for cardiac regeneration in neonates. Postnatal day one (P1) LPA_3_ KO mice underwent myocardial infarction (MI) by coronary artery occlusion and were assessed after 4, 7, and 21 days (Figure 2A). The results showed that cardiac function was decreased in the LPA_3_ KO mice compared to the wild-type controls (Figure 2B), consistent with the increased scar size determined using picrosirius red staining (Figure 2C). Correspondingly, cardiomyocyte proliferation, as assessed by the percentages of pH3-, Ki67- and BrdU-positive cardiomyocytes, was reduced in the LPA_3_ KO mice at both P4 and P7 (Figure 2D).

Adeno-associated virus 9 (AAV9) is a safe and cardiotropic vector that is widely used in the myocardium for gene modification. Thus, we generated AAV9-cTNT- 3Flag: LPA_3_ (AAV-LPA_3_), in which the cardiomyocyte-specific cardiac troponin T (cTNT) promoter drives the expression of 3Flag-tagged LPA_3_ in the heart. AAV9-cTNT-EGFP was generated as a control. We first validated the efficiency of LPA_3_ expression driven by the viral vector by delivering these vectors subcutaneously to P1 mice (Figure S2A). Seven days or 28 days after virus injection, the expression of LPA_3_ was analyzed by Western blots, qRT-PCR and immunofluorescence. Western blot analysis showed a Flag-labeled LPA_3_ band in the AAV9-LPA_3_-treated mice, and qRT-PCR showed that LPA_3_ was overexpressed at both P7 and P28 compared to AAV-EGFP (Figure S2B and C). Immunofluorescence staining for Flag demonstrated strong and specific membrane expression of LPA_3_ on the cardiomyocytes of the AAV-LPA_3_ group (Figure S2D).

**Figure 3:**
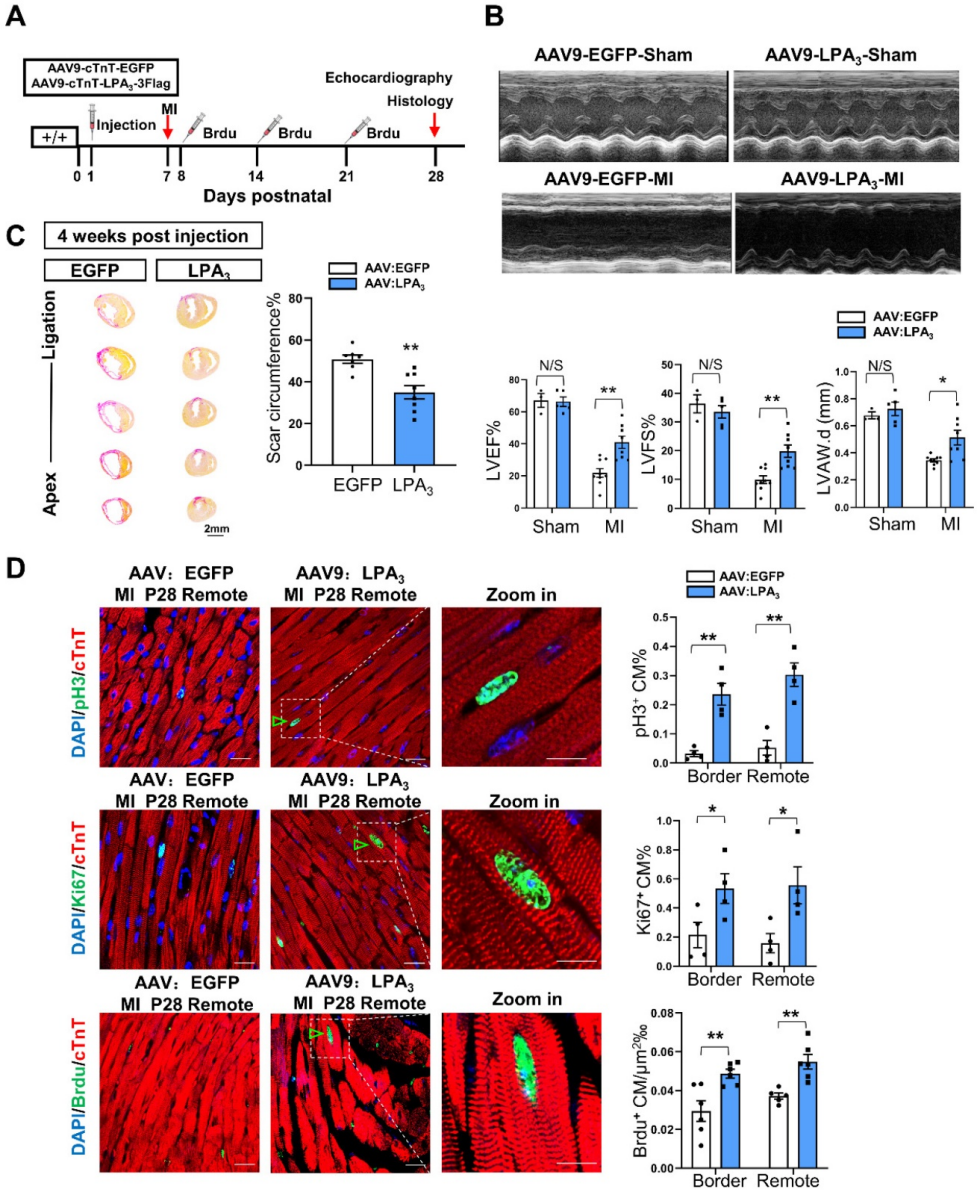
** Cardiac-specific overexpression of LPA_3_ enhances cardiac function and regeneration in neonates after myocardial infarction. (A)** Experimental design and timeline of cardiac-specific overexpression of LPA_3_ in the neonatal heart. **(B)** Echocardiography assessment of cardiac function of the AAV9:LPA_3_ and AAV9:EGFP mice at 21 days after MI on P7 (sham n = 3-5, MI n = 9 per group). LVAW.d, left ventricular anterior wall in diastole. **(C)** Scale size of the AAV9:LPA_3_ and AAV9:EGFP mice after MI (n = 8-10 per group, scale bar = 2 mm). **(D)** Immunofluorescence and quantification of the pH3-, Ki67-, and BrdU-positive cardiomyocytes at day 21 after surgery in the AAV9:LPA_3_ and AAV9:EGFP mice (n = 4-6 per group, scale bar of the images = 20 µm; scale bar of the close-up image =10 µm). Green arrowheads indicate proliferating cardiomyocytes. Data are the mean ± SEM; nonsignificant (N/S), *P* > 0.05; “n” indicates the number of mice; each point in the scatter plot indicates the data of individual mice; *P< 0.05; **P<0.01.

We next examined the potential therapeutic function of AAV9-LPA_3_ in the heart. MI was induced on P7 and assessed 21 days later in the mice with cardiac-specific LPA_3_ overexpression by subcutaneous injection of AAV9-LPA_3_ at P1 (Figure 3A). In these mice, we found that left ventricular systolic function improved significantly 21 days after MI (Figure 3B). This finding is consistent with a decrease in the scar size (Figure 3C). Moreover, the percentages of pH3-, Ki67- and BrdU-positive cardiomyocytes increased in both the border and remote areas, in contrast to those of the control mice (Figure 3D). In addition, we investigated whether overexpression of LPA_3_ has an effect on the healthy heart. The results showed that overexpression of LPA_3_ had no effect on cardiomyocyte proliferation in the healthy hearts of P8 mice, as shown by the Ki67- and pH3-positive cardiomyocyte percentages (Figure S3A). Moreover, overexpression of LPA_3_ did not cause cardiac hypertrophy, as indicated by the heart size and heart-to-body weight ratio (Figure S3B). Taken together, these results demonstrate that LPA_3_-mediated LPA signaling is necessary for cardiac regeneration in neonatal mice by promoting cardiomyocyte proliferation.

### Cardiac-specific overexpression of LPA_3_ enhances cardiac function and increases cardiomyocyte proliferation after myocardial infarction in adult mice

**Figure 4:**
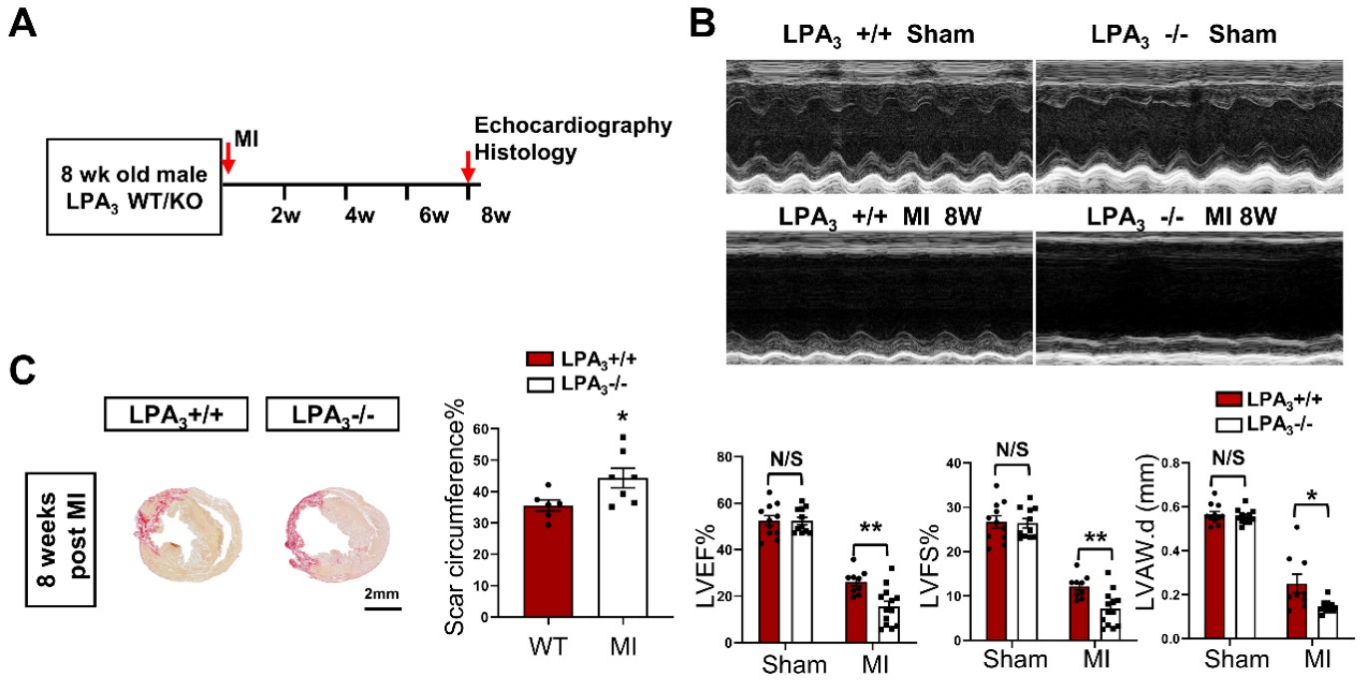
** Cardiac function decrease in the adult LPA_3_ KO mice after myocardial infarction. (A)** Experimental design and timeline of MI in the adult mice.** (B)** Echocardiography assessment of cardiac function of the adult LPA_3_ WT and KO mice 8 weeks after MI (n = 9-11 per group). **(C)** Scale size of the LPA_3_ WT and KO adult mice after MI (n = 5 per group, scale bar = 2 mm). Data are the mean ± SEM; nonsignificant (N/S), *P* > 0.05; “n” indicates the number of mice; each point in the scatter plot indicates the data of individual mice; *P < 0.05, **P<0.01.

We next examined whether LPA_3_-mediated LPA signaling could promote cardiomyocyte proliferation and cardiac regeneration in adult mice. First, cardiac function and myocardial injury were assessed after MI in 16-week-old mice (Figure 4A). The results showed a decrease in cardiac function, as measured by echocardiography and shown by the LVEF, LVFS and LVAW, in the LPA_3_ KO mice compared with the controls (Figure 4B). In addition to decreased cardiac function, there was an increase in the scar size in the hearts of the LPA_3_ KO mice after MI (Figure 4C). However, there were no significant changes in cardiomyocyte apoptosis as assessed by TUNEL assays between the LPA_3_ KO mice and the wild-type controls after MI (Figure S1B). These results suggest that the myocardium in the LPA_3_ knockout adult mice may be more vulnerable in response to injury due to the decreased cardiomyocyte number, as mentioned before.

**Figure 5:**
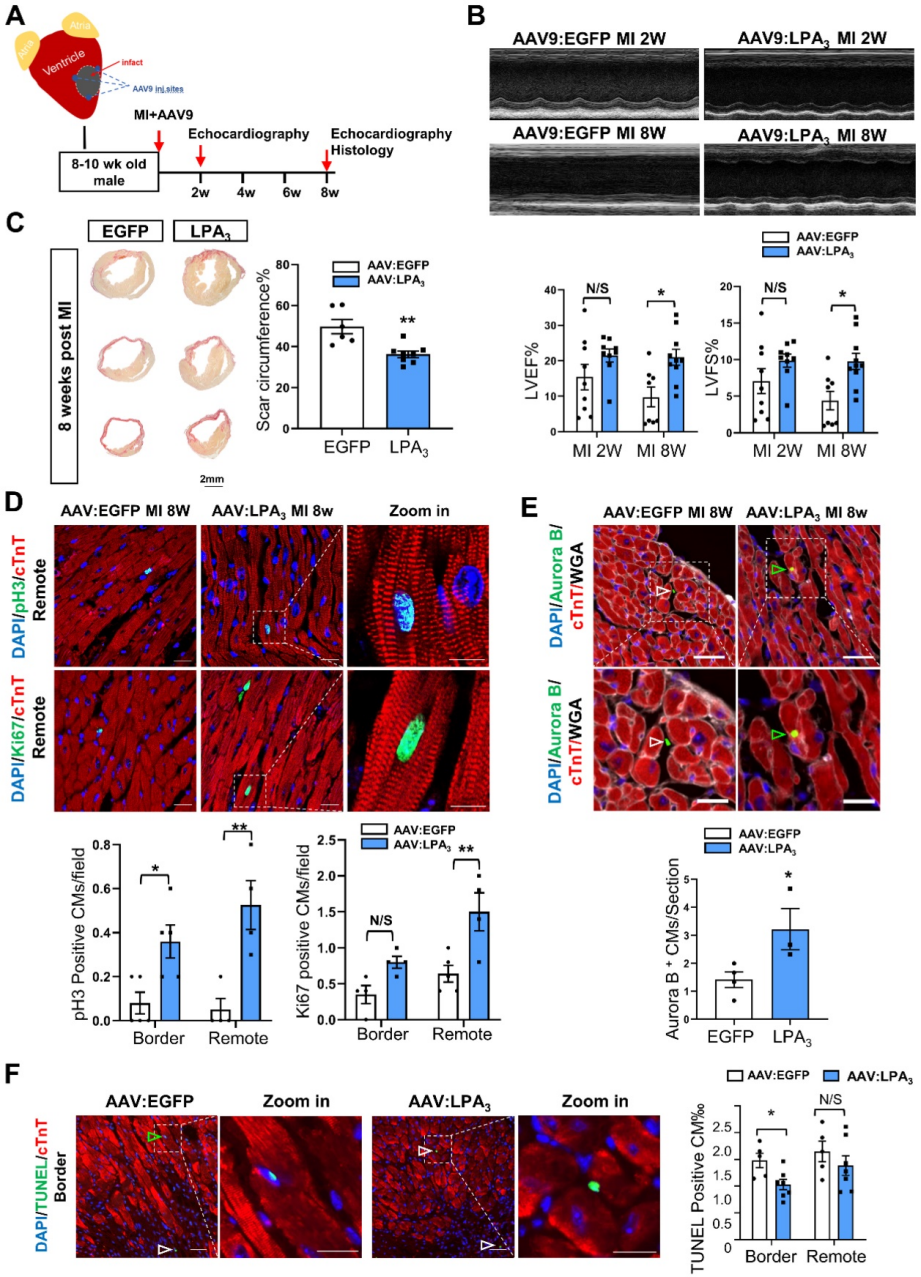
** Cardiac-specific overexpression of LPA_3_ enhances cardiac function and increases cardiomyocyte proliferation after myocardial infarction in adult mice. (A)** Schematic of the AAV9:LPA_3_ therapeutic trial with wild-type mice.** (B)** Echocardiography assessment of cardiac function at 2 weeks and 8 weeks after MI in the AAV9:LPA_3_ and control mice (n = 8-10 per group)**. (C)** Scale size of the adult AAV9:LPA_3_ and AAV9:EGFP mice at 8 weeks after MI (n = 6-8 per group, scale bar = 2 mm).** (D)** pH3 and Ki67 immunofluorescence of cardiomyocytes in the AAV9:LPA_3_ and control adult mice at 8 weeks after MI (n = 5 per group). Scale bar of the images = 20 µm; scale bar of the close-up image = 10 µm. **(E)** Aurora B immunofluorescence of cardiomyocytes in the AAV9:LPA_3_ and control adult mice at 8 weeks after MI (n=4 per group). Scale bar of the upper images = 20 µm; scale bar of the lower images = 10 µm. Yellow arrows indicate Aurora B-positive cardiomyocytes; white arrows indicate Aurora B-positive noncardiomyocytes. **(F)** TUNEL immunofluorescence of cardiomyocytes of the adult AAV9:LPA_3_ and control mice at 8 weeks after MI (n=5 per group). Green arrowheads indicate apoptotic cardiomyocytes; white arrowheads indicate apoptotic noncardiomyocytes. Scale bar of the images = 20 µm; scale bar of the close-up image = 10 µm. Data are the mean ± SEM; nonsignificant (N/S), “n” indicates the number of mice; each point in the scatter plot indicates the data of individual mice. *P* > 0.05; *P < 0.05; **P<0.01.

Next, to further examine whether LPA_3_-mediated signaling promotes cardiomyocyte proliferation and cardiac regeneration in adult mice, we injected the mice with AAV9-LPA_3_ or AAV9-EGFP in the heart immediately after ligation of the left anterior descending artery (Figure 5A). Cardiac-specific overexpression of LPA_3_ improved cardiac function at 2 weeks after MI, with a significant improvement at 8 weeks after MI, as indicated by echocardiography (Figure 5B). Histological analysis revealed that the scar area was reduced in the LPA_3_-overexpressing hearts (Figure 5C). Furthermore, coimmunostaining with anti-pH3 or anti-Ki67 and anti-cTnT antibodies showed significantly increased cardiomyocyte proliferation in the infarcted hearts from the mice overexpressing LPA_3_ (Figure 5D). Cardiomyocyte mitosis was also assessed by the number of Aurora B-positive cardiomyocytes, which showed a significant increase in the LPA_3_-overexpressing hearts (Figure 5E). Furthermore, TUNEL assays showed that the apoptosis of cardiomyocytes in the border zone decreased in the LPA_3_-overexpressing hearts compared with the controls (Figure 5F).

**Figure 6:**
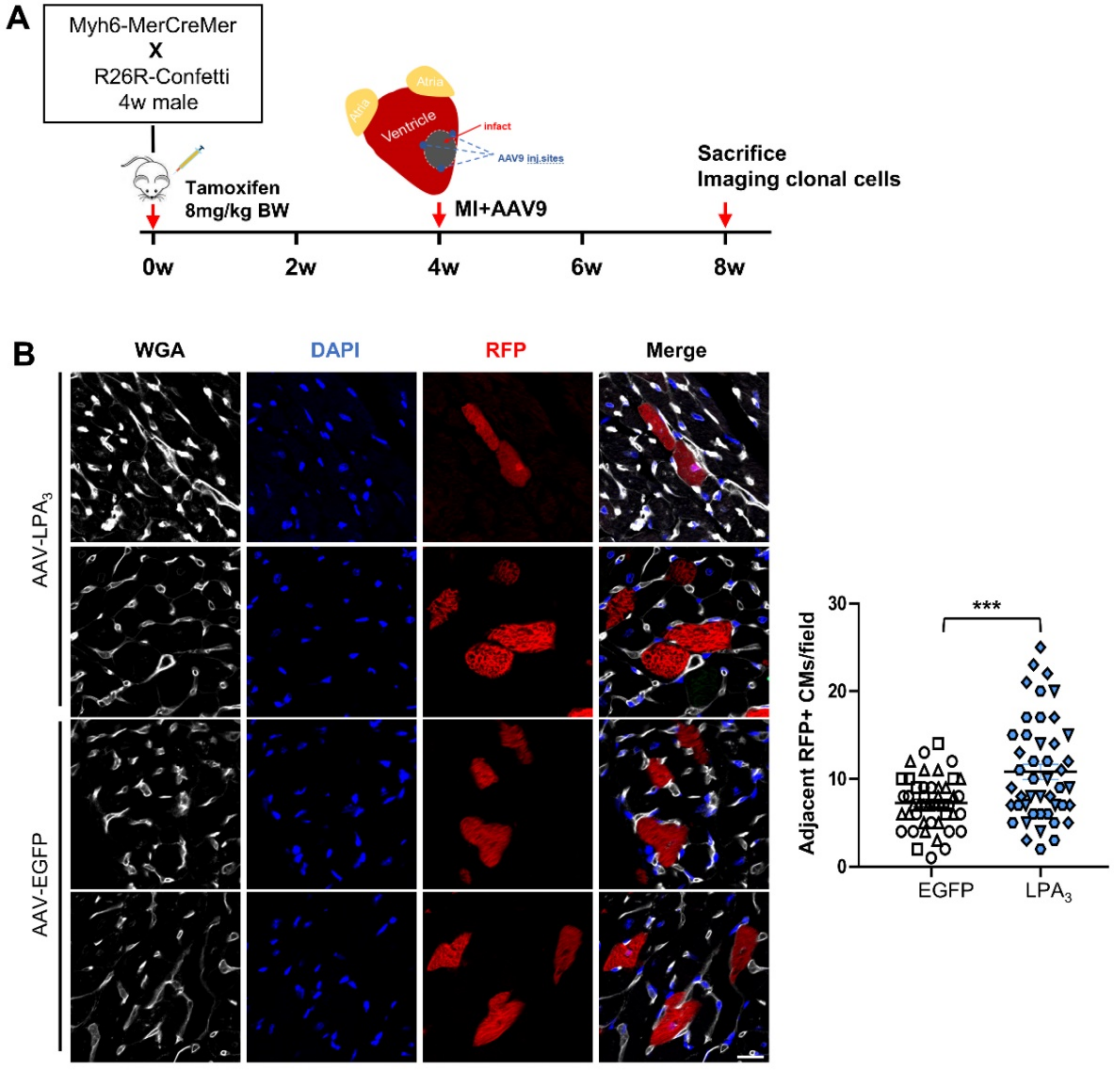
** LPA_3_ overexpression induces clonal expansion in the adult hearts after MI. (A)** Schematic of tamoxifen induction and experimental design. **(B)** Representative image of cardiomyocytes expressing RFP and quantification of the adjacent RFP-positive cardiomyocyte clones. Scale bar = 20 µm, Data are the mean ± SEM; AAV-EGFP, n = 3; AAV-LPA_3_, n = 3; each point in the scatter plot indicates the data of individual fields captured from 3 mice of each group. “n” indicates the number of the mice. Different symbol shapes indicate each animal; ***P < 0.001.

To verify that the proliferation observed after injection of AAV9-LPA_3_ in the heart indeed generated new cardiomyocytes after MI, we performed lineage tracing using a multicolor R26R-Confetti Cre-reporter system (Figure 6A). In addition to fluorescent reporters, we used wheat germ agglutinin (WGA) staining to identify cell membranes, which separated individual cardiomyocytes and enabled identification of clones. In the AAV9-LPA_3_ heart, we observed multiple clones in close proximity, consisting of daughter cells from a single parent cell. In contrast, the control group consisted mostly of individual cardiomyocytes that were spatially separated (Figure 6B). By quantifying the number of clonal cardiomyocytes expressing red fluorescent protein (RFP), we found that the AAV9-LPA_3_ hearts had a significant increase in the number of clones per section, suggesting increased lineage-labeled cardiomyocytes due to clonal expansion (Figure 6C). These results indicate that cardiac-specific overexpression of LPA_3_ enhances cardiac function and promotes cardiomyocyte proliferation after MI in adult mice.

### LPA promotes cardiomyocyte proliferation *in vitro* through LPA_3_

**Figure 7:**
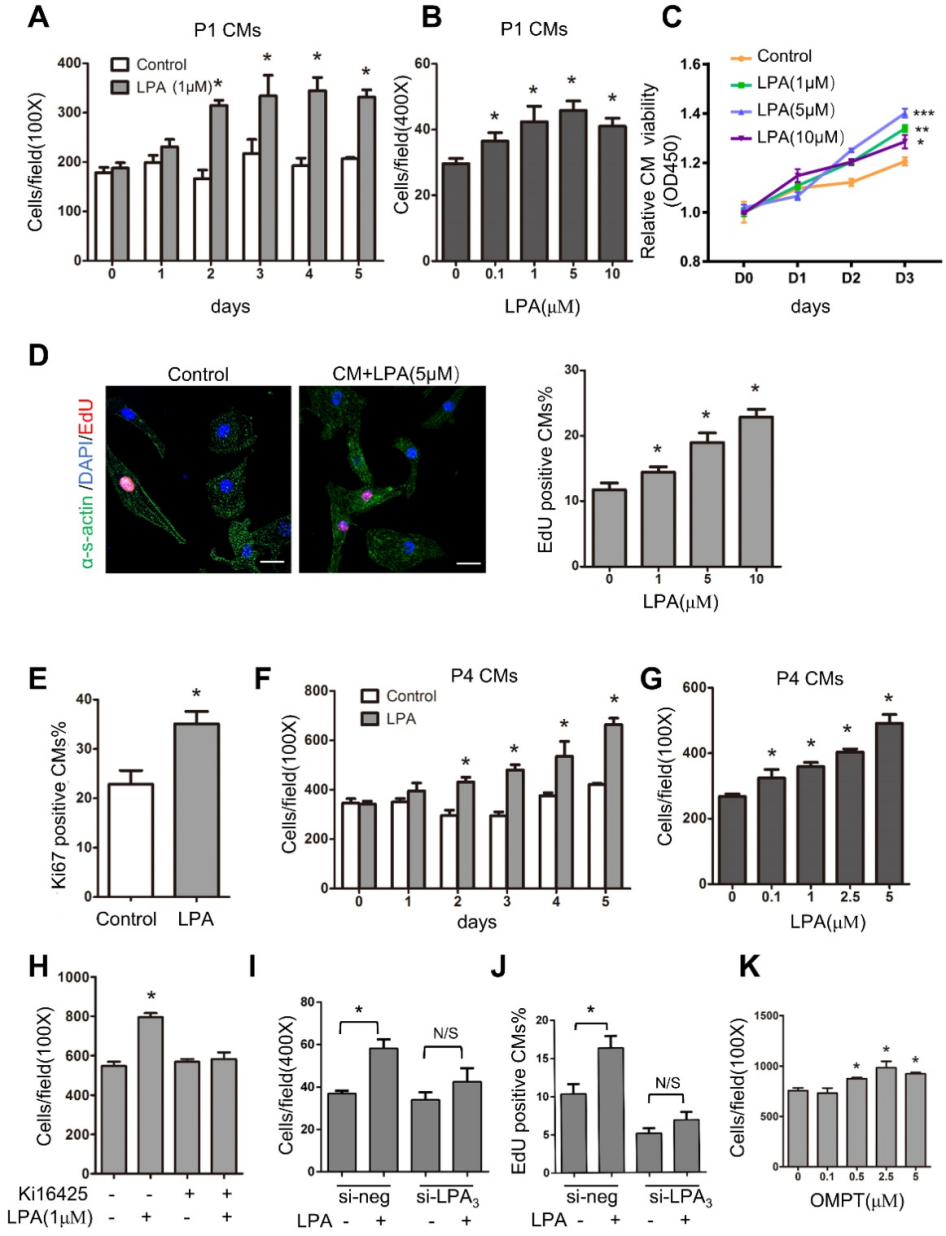
** LPA promotes cardiomyocyte proliferation *in vitro* through LPA_3_**. **(A)** Cardiomyocytes isolated from postnatal day 1 rat hearts (P1 CMs) were treated with 1 µM LPA for 1-5 days, and the number of cardiomyocytes was counted. **(B)** P1 CMs were treated with LPA at the indicated concentrations, and the number of cardiomyocytes was counted. **(C)** The CCK-8 assay was used to evaluate the proliferation of neonatal cardiomyocytes after treatment with different concentrations of LPA. **(D)** Representative images of EdU and quantification of EdU-positive cardiomyocytes after treatment with LPA at different concentrations. (Scale bar = 20 µm). **(E)** Quantification of the Ki67-positive cardiomyocytes after treatment with 1 µM LPA. **(F)** Cardiomyocytes isolated from the postnatal day 4 rat hearts (P4 CMs) were treated with 1 µM LPA for 1-5 days and then counted. **(G)** P4 CMs were treated with LPA at the indicated concentrations for 48 h and then counted. **(H)** P1 CMs were treated with Ki16425 before LPA, and the number of cardiomyocytes was counted.** (I, J)** P1 CMs were transfected with siRNAs targeting LPA_3_ (si-LPA_3_) or negative control (si-NEG) before LPA treatment, and the total **(I)** and EdU-positive **(J)** cardiomyocytes were counted. **(K)** P1 CMs were treated with OMPT (LPA_3_ agonist) at the indicated concentrations, and the number of cardiomyocytes was counted. n = 3 samples of each group; data are presented as the mean ± SEM; *P<0.05.

To identify the role of LPA in immature cardiomyocyte proliferation *in vitro*, we first used cardiomyocytes isolated from P1 rats. These cells actively divide, and the proliferation rate can be measured. Cardiomyocytes were treated with LPA at different concentrations (0.1, 1, 5 and 10 μM) for 1-5 days, and the total number of cardiomyocytes (marked by α-sarcomeric actin) was counted to evaluate cardiomyocyte proliferation. As shown in Figure 7A-B, LPA induced cardiomyocyte proliferation in a time- and concentration-dependent manner. Moreover, cardiomyocyte proliferation was measured by Cell Counting Kit-8 (CCK-8), and the results showed that cell viability increased significantly with an increase in LPA concentration (1, 5 and 10 μM) and culture duration (Day 0~Day 3) (Figure 7C). In addition, using an EdU incorporation assay, we found that LPA treatment (1, 5 and 10 μM) increased the number of EdU-positive cardiomyocytes in a concentration-dependent manner (Figure 7D). Furthermore, the number of Ki67-positive cardiomyocytes increased significantly upon treatment with LPA (1 μM) for 48 h (Figure 7E). We then treated postnatal day 4 (P4) cardiomyocytes, which showed a decrease in proliferative potential, with LPA. We found that LPA also promoted P4 cardiomyocyte proliferation in a time- and concentration-dependent manner (Figure 7F-G). Together, our data demonstrate that LPA can stimulate the proliferation of cardiomyocytes *in vitro*.

We used the LPA_1_/LPA_3_ antagonist Ki16425 and found that it abolished the LPA-induced increase in cardiomyocyte number (Figure 7H). In addition to pharmacological inhibitors, we employed LPA_3_ siRNA and found that the LPA-induced cardiomyocyte proliferation was substantially inhibited when LPA_3_ was depleted by siRNA knockdown (Figure 7I-J). qRT-PCR confirmed the efficiency of si-LPA_3_-mediated LPA_3_ knockdown (Figure S4A). However, the LPA_3_ agonist 1-oleoyl-2-O-methyl-rac-glycero-phosphothionate (OMPT) significantly stimulated cardiomyocyte proliferation at concentrations ranging from 0.5 μM to 5 μM (Figure 7K). These data demonstrated that LPA promotes immature cardiomyocyte proliferation through LPA_3_ *in vitro*.

### LPA_3_-mediated LPA signaling activates ERK to induce cardiomyocyte proliferation

**Figure 8:**
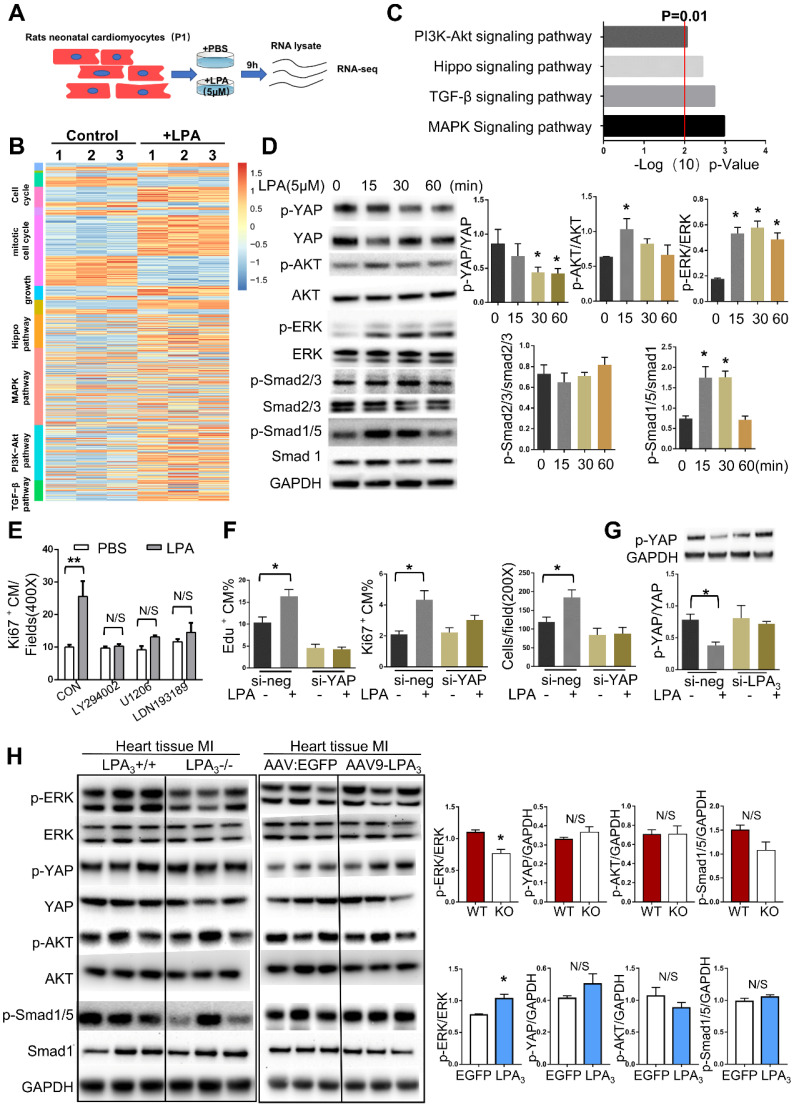
** LPA_3_-mediated LPA signaling activates ERK to induce cardiomyocyte proliferation. (A)** Schematic of total RNA-seq**. (B)** Heatmap of proliferation-associated Gene Ontology (GO) and KEGG pathways**. (C)** KEGG pathway analysis of total RNA-seq.** (D)** P1 rat cardiomyocytes were treated with 5 µM LPA for the indicated times. The expression of p-YAP, p-AKT, p-ERK, p-Smad2/3, p-Smad1/5, their unphosphorylated counterparts, and GAPDH were detected by Western blots and quantified. **(E)** The percentages of Ki67-positive cardiomyocytes induced by LPA after treatment with different inhibitors of the signaling pathways tested above (LY294002, inhibitor of PI3K; U1206, inhibitor of ERK; LDN193189 inhibitor of BMP signaling pathway). **(F)** The total number of cardiomyocytes and the percentages of Ki67- and EdU-positive cardiomyocytes induced by LPA after YAP1 knockdown, as evaluated by immunostaining.** (G)** P1 rat cardiomyocytes were transfected with si-LPA_3_ or negative control. Two days later, the cells were treated with or without 1 µM LPA for 30 min. p-YAP and GAPDH were detected by Western blots and quantified. **(H)** Protein from the LV was isolated 2 days after MI in the LPA_3_ WT and KO mice or 21 days after MI in the AAV9:LPA_3_ and AAV9:EGFP mice. The expression of p-YAP, p-AKT, p-ERK, p-smad1/5, ERK and GAPDH was detected by Western blots and quantified. Data are presented as the mean ± SEM; n = 3 samples of each group; nonsignificant (N/S), *P* > 0.05; *P<0.05; **P<0.01.

Since we found that different concentrations of LPA ranging from 0.1 μM to 10 μM could induce cardiomyocyte proliferation and that 5 μM LPA had the most significant effect, we next used 5 μM LPA to explore the potential mechanism involved in LPA promotion of cardiomyocyte proliferation. To gain a greater understanding of the effect of LPA on cardiomyocyte proliferation, we performed genome-wide transcriptional profiling on cardiomyocytes treated with or without 5 μM LPA and found distinctive sets of genes that were regulated by LPA (Figure 8A). Genes upregulated by LPA were related to the cell cycle, cell growth and several proliferation-related signaling pathways (Figure 8B). By performing KEGG pathway analysis, we found that several signaling pathways, including the PI3K/AKT, Hippo, TGF-beta and MAPK pathways, were enriched (Figure 8C).

To further confirm the key signaling events affected by LPA treatment in cardiomyocytes, we performed Western blots and observed rapid YAP dephosphorylation and phosphorylation of AKT, ERK, and Smad1/5 but not Smad2/3 (Figure 8D). These results indicate that LPA activates the Hippo, PI3K/AKT, MAPK/ERK, and BMP-Smad1/5/8 signaling pathways but not the TGFβ-Smad2/3 signaling pathway. As a complementary set of experiments, by blocking the BMP-Smad1/5/8, AKT, and ERK pathways with LDN-193189, LY294002 and U1206, respectively, we found that LPA-induced cardiomyocyte proliferation was completely abrogated by the three inhibitors (Figure 8E). Considering that LPA was recently identified as an extracellular diffusible signal that modulates the Hippo/Yap pathway 15, we further focused on the YAP signaling pathway. This recently discovered signaling pathway regulates organ growth 16 and has potent effects on cardiomyocyte proliferation 17. To test whether LPA induces cardiomyocyte proliferation by activating YAP, we depleted YAP1 using siRNA. YAP1 knockdown resulted in a significant reduction in the expression of YAP (Figure S4B) and prevented the increase in the total number of cardiomyocytes and the percentage of Ki67- and EdU-positive cardiomyocytes induced by LPA (Figure 8F). In addition, LPA-induced YAP dephosphorylation was blocked by LPA_3_ siRNA (Figure 8G). These data suggest that LPA may induce cardiomyocyte proliferation by activating downstream signaling molecules, including AKT, ERK, Smad1/5, and YAP,* in vitro*.

However, when these signaling molecules were tested in mouse hearts after MI, we found that only the ERK pathway was downregulated in the LPA_3_ KO mice and activated in the LPA_3_-overexpressing mice (Figure 8H). Therefore, based on the results from both *in vitro* and *in vivo* experiments, we conclude that LPA_3_-mediated LPA signaling may activate ERK to induce cardiomyocyte proliferation.

## Discussion

LPA signaling plays essential roles in many developmental processes, modulating a number of organ systems and cell types 18. In this study, we reveal a previously unknown role of LPA signaling in the regulation of cardiomyocyte proliferation in the early postnatal period. More importantly, we found that LPA_3_-mediated LPA signaling is necessary for cardiac regeneration in neonatal mice and cardiac repair in response to injury in adult mice.

Although the mammalian heart has long been considered a postmitotic organ, in the past several years, extensive reports have confirmed the generation of new cardiomyocytes in mouse and human hearts after birth 9-11, 19. However, the underlying mechanism of cardiomyocyte proliferation after birth remains largely unclear. It has been reported that some proteins and microRNAs, such as Neuregulin 1 14, agrin 20, miRNA-17-92 21 and miRNA-708 22, regulate postnatal cardiomyocyte proliferation. Here, we propose that the lipid LPA signaling pathway can promote cardiomyocyte proliferation after birth. We found that the LPA_3_ KO mice exhibited a decrease in proliferating cardiomyocytes during the first week after birth and a 30% decrease in the total number of cardiomyocytes in the hearts of the adult mice compared with the wild-type controls. The percentage of this decrease is consistent with the percentage of newly generated cardiomyocytes in this stage, as reported by others 8, 23, 24. Li et al. stated that the newly generated cardiomyocytes during the first 3 days contributed to approximately 40% of the total number of adult cardiomyocytes 8. Two other published recently studies also found that the proliferation of cardiomyocytes during the first 4 to 5 days after birth increases cardiomyocyte numbers by approximately 40% 23, 24. These results suggest that LPA_3_-mediated LPA signaling may play an important role during this period of cardiac growth between birth and adolescence.

Although the adult mammalian heart has the potential for regeneration, it is not enough to compensate for the loss of cardiomyocytes during injury and disease. The regulatory mechanisms involved in heart growth and development can be explored to repair the injured adult heart by 'reawakening' signaling pathways active during early developmental stages 25. Encouragingly, we found that this strategy works for LPA_3_-mediated LPA signaling. Our study showed that cardiac-specific overexpression of LPA_3_ enhanced cardiac function and promoted regeneration after MI in not only neonatal but also adult mice. This finding implies that activation of LPA_3_-mediated LPA signaling, which is necessary for cardiomyocyte proliferation during the early postnatal period, could serve as a potential therapeutic option for cardiac repair in adults.

Although it has been demonstrated that LPA signaling influences many cell types, few studies have reported the biological effect of LPA on cardiomyocytes. Hilal-Dandan et al. 26 and our previous study 27 have described LPA-induced hypertrophy in neonatal rat cardiomyocytes *in vitro*. Our further *in vivo* study found that LPA_3_ deficiency attenuated cardiac hypertrophy but aggravated cardiac dysfunction after MI 28. In this paper, we showed that LPA-LPA_3_ signaling promotes cardiomyocyte proliferation both *in vitro* and *in vivo*. We speculate that LPA-LPA_3_ signaling may play a stage-specific role in the heart. LPA-induced cardiomyocyte proliferation might contribute to heart growth largely during the early postnatal period, and LPA-induced cardiomyocyte enlargement might be responsible for the compensatory cardiac hypertrophy involved in myocardial remodeling. It is believed that LPA signaling acts as a 'bad' modulator in pathological cardiovascular responses 6. However, emerging evidence has established a protective role of LPA signaling in heart disease. For example, the delivery of LPA-treated CD34^+^ cells to the infarcted heart improved cardiac function 29. LPA-LPA_3_ protected cardiomyocytes from hypoxia/reperfusion-induced injury by suppressing the mitochondrial apoptotic pathway 30. In our present study, the results indicated that cardiac-specific overexpression of LPA_3_ enhances cardiac function after MI. Although loss of LPA_3_ did not affect the apoptosis of cardiomyocytes during the first week, cardiac-specific overexpression of LPA_3_ promoted the survival of cardiomyocytes after MI. It may be the other side of coin.

In addition to providing compelling data that demonstrate the importance of LPA signaling in promoting heart regeneration, our study also raises new questions. Several studies have reported the proatherosclerotic, proinflammatory and proangiogenic effects of LPA signaling by influencing many cell types, such as immune cells, endothelial cells, and fibroblasts 2, 18, 31. Recently, Das et al. proposed a connection between collateral artery development and neonatal heart regeneration 32. In addition, different studies have consistently shown that macrophages are required for neonatal heart regeneration and cardiac repair 33, 34. Therefore, these studies indicated that LPA signaling may have other beneficial effects in the process of cardiac repair proliferation through different kinds of cells in the heart in addition to cardiomyocytes. In our present study, by using LPA_3_ knockout mice and an AAV9-mediated cardiac-specific LPA_3_ expression model, we shed light on the role of LPA_3_-mediated LPA signaling in cardiac regeneration by controlling cardiomyocyte proliferation. Indeed, there are some limitations of using LPA_3_ whole-body knockout mice to conclude that LPA_3_-mediated LPA signaling influences cardiac regeneration through effects on cardiomyocytes. Thus, we generated AAV9-cTNT-3Flag:LPA_3_ to examine whether cardiac-specific overexpression of LPA_3_ after myocardial injury improved heart function and promoted cardiomyocyte proliferation. The results showed that cardiac-specific activation of LPA_3_ drives cardiomyocyte proliferation and improves the outcome after MI. However, it is difficult to exclude the function of LPA signaling on other kinds of cells, which was proven to be involved in cardiac regeneration. Thus, cardiac-specific knockout mice of LPA_3_ should be applied in future research to generate a more powerful conclusion. LPA signaling might have pleiotropic effects on cardiac repair in response to injury, which warrants further investigation.

In summary, our study reports for the first time that a lipid molecule, LPA, modulates cardiomyocyte proliferation in the early postnatal heart. Furthermore, this signaling pathway is essential for cardiac repair and regeneration in response to injury in adult rodents. Importantly, LPA receptors are part of a large class of GPCR drug targets, raising the possibility of potentially treating some conditions impacted by LPA signaling by targeting its receptors.

## Materials and Methods

### Animals

The LPA_3_- and LPA_1_-knockout mice were gifts from Professor Jerold Chun 35, 36. The primer sequences used for LPA_1_ and LPA_3_ knockout mouse genotype identification are listed in Table 1. Sprague-Dawley (SD) rats and Balb/c mice were obtained from Vital River Laboratory Animal, Inc. (China). Brainbow2.1 lineage mice were donated by Professor Wang Dazhi at Harvard University's Children's Hospital, Boston. Animal experiments were approved by the Ethics Committee for Animal Study in Fu Wai Hospital (animal application approval number 2012-5-30-973). The investigation conforms to the Guide for the Care and Use of Laboratory Animals published by the US National Institutes of Health (NIH Publication No. 85-23, revised 1996) and the 'Regulation to the Care and Use of Experimental Animals' of the Beijing Council on Animal Care (1996).

### Myocardial infarction in neonatal mice

MI surgeries were performed on the LPA_3_ WT and KO neonatal mice at P1. Neonates were anesthetized by cooling on an ice bed for 2 min. Lateral thoracotomy at the fourth intercostal space was performed by blunt dissection of the intercostal muscles after skin incision. A tapered needle (C-1) attached to a 6-0 prolene suture (Ethicon) was passed through the mid-ventricle below the origin of the left anterior descending (LAD) coronary artery and tied to induce infarction. The pericardial membrane remained intact after LAD ligation. Myocardial ischemia was indicated by the light pallor of the myocardium below the ligature after suturing. After LAD ligation, the neonates were removed from the ice bed, and thoracic wall incisions were sutured with a 6-0 nonabsorbable prolene suture. Sham-operated mice underwent the same procedure involving hypothermic anesthesia and thoracotomy without LAD ligation.

### AAV9 packaging

AAV9 packaging was performed by OBiO Technology (Shanghai) Corp. Briefly, 3Flag-LPA_3_ and EGFP were separately cloned into ITR-containing AAV plasmids (Penn Vector Core P1967) harboring the chicken cardiac TNT promoter to obtain pAAV.cTnT::3Flag-LPA_3_ and pAAV.cTnT::EGFP, respectively. AAV9 was packaged in 293T cells.

### Construction of cardiac-specific LPA_3_ overexpression neonatal mice

Neonatal mice at P1-P2 were subjected to subcutaneous injection in the back. Each mouse was injected with 1 × 10^11^ vg in a 10 μL final volume in phosphate-buffered saline (PBS) of either AAV9:LPA_3_ or control AAV:EGFP.

### Myocardial infarction in adult mice

MI was induced by ligation of the left anterior descending coronary artery. LPA_3_ KO and WT mice at 8-10 weeks were used for the infarction experiment. This MI model was generated as previously described 37. Mice were anesthetized with tribromoethanol (400 mg/kg; IP) and ventilated with a rodent respirator. Then, the LAD coronary artery was permanently occluded using a 7-0 polypropylene suture, and the occlusion was confirmed by blanching of the anterior wall of the left ventricle. As noninfarcted controls, mice underwent a sham operation where the ligature around the LAD was not tied. The animals recovered from anesthesia under warm conditions with normal ventilation. Eight weeks after surgery, the animals were sacrificed, and the hearts were excised for further analysis.

For cardiac-specific expression of LPA_3_ in adult mice after MI, virus was injected directly into the myocardium at three positions along the margin of the ischemic area when performing the MI. Each mouse was injected with 1 × 10^11^ vg in a 20 μL final volume in phosphate-buffered saline (PBS) of either AAV9:LPA_3_ or control AAV:EGFP.

### Ki16425 treatments

Ki16425 (Cayman, USA) powder was first dissolved in DMSO at a concentration of 100 μg/μL and then diluted in PBS to a final concentration of 5 μg/μL. Ki16425 (20 mg/kg) and vehicle (control) were administered by intraperitoneal injections daily from the day of birth 38. For 5-bromo-2-deoxyuridine (BrdU, Sigma, USA) labeling experiments, BrdU pulse-chase was performed according to a published protocol 9.

### Lineage tracing

To clarify the effect of LPA-LPA_3_ signaling on myocardial regeneration after myocardial infarction, we performed lineage tracing analysis using Brainbow2.1 lineage mice. Brainbow2.1 mice were constructed with loxP-flanked nuclear green fluorescent protein (nGFP), red fluorescent protein (RFP), yellow fluorescent protein (YFP) and monomeric cyan fluorescent protein (mCFP). When bred with the inducible Cre transgene expressed under the myosin heavy chain 6 promoter, the Brainbow2.1 lineage mice would express only one of the four fluorescent protein genes randomly in the cardiomyocytes after intraperitoneal tamoxifen injection. A suitable dose of tamoxifen used ensures low levels of cardiomyocyte labeling so that two adjacent cardiomyocytes expressing the same gene can be considered as a clone. Briefly, intraperitoneal tamoxifen (9 mg/kg BM) single injection was performed when Brainbow2.1 lineage male mice were one month old. Four weeks after tamoxifen injection, myocardial infarction was conducted as mentioned above, and virus was injected directly into the myocardium at three positions along the margin of the ischemic area. Each mouse was injected with 1 × 10^11^ vg in a 20 μL final volume in phosphate-buffered saline (PBS) of either AAV9:LPA_3_ or control AAV:EGFP. Four weeks after myocardial infarction, the mice were sacrificed, and the hearts were fixed overnight with 10% formalin. After 24 h, the hearts were transferred to 3% sucrose solution for 24 h, embedded in OCT, frozen at -80°C and sectioned. After WGA/DAPI staining, the sections were imaged to observe the fluorescent protein expressed by myocardial cells. Adjacent RFP-positive cardiomyocytes were counted.

### Echocardiography

Echocardiographic measurements were performed using a VisualSonics Vevo 770 High Resolution Imaging System (Visual Sonics, Canada) with 40 MHz and 30 MHz MicroScan transducers. Fractional shortening (FS) and the ejection faction (EF) were calculated based on end diastolic and end systolic dimensions obtained from M-mode ultrasound.

### Counting of adult cardiomyocytes

This method of adult cardiomyocyte isolation and counting has been described elsewhere 39. Briefly, hearts were harvested, fixed in 4% paraformaldehyde (PFA) at a temperature of 4°C overnight (the atria were removed before fixation) and then digested with collagenase D (2.4 mg/mL, Roche) and B (1.8 mg/mL, Roche) for 12 h at 37°C. The supernatant was collected, and the cardiomyocytes were centrifuged. The hearts were minced into smaller pieces, and the above procedure was repeated until no more cardiomyocytes were dissociated from the tissue. Finally, rod-shaped cells were counted using a hemocytometer.

### Cardiomyocyte isolation and culture

Neonatal cardiomyocytes were isolated as previously described from 1- or 4-day-old (P1 or P4) SD rats 27. Cardiomyocytes were then cultured in DMEM containing 10% fetal bovine serum, penicillin/streptomycin (1000 U/mL each), and 100 mM BrdU to inhibit the growth of the cardiac fibroblasts. Twenty-four hours after plating, the cells were starved in serum-free medium overnight. Then, the cells were stimulated with LPA for different lengths of time in serum-free medium. Ki16425 treatment was performed 1 h before LPA exposure. For transfection experiments, 100 nM siRNA and negative control siRNA were transfected into cardiomyocytes using Lipofectamine 2000 transfection reagent (Invitrogen, USA). Stimulation experiments were performed after 24 h of transfection. EdU (5-ethynyl-2'-deoxyuridine, Invitrogen, USA) was added 24 h before the cells were fixed for immunofluorescence analyses. For the cell viability assay, Cell Counting Kit-8 (CCK-8, Beyotime, China) solution was added 2 h before the cells were measured at 450 nm.

### Histology and immunofluorescence

Whole hearts were fixed in 10% formalin solution for 24 h and embedded in paraffin. Hearts were cut longitudinally into 5 μm sections. Each heart had 6~7 sections, which started at the apex and ended at the ligation site. For infarct size measurement, paraffin-embedded sections (5 μm) were prepared, and scar circumference was calculated using picrosirius red staining and presented as the average of serial sections from the apex to the ligation. Hematoxylin/eosin staining was performed according to standard laboratory procedures.

All immunofluorescence analyses were performed on PFA-fixed, paraffin-embedded sections. After deparaffinization and rehydration, the sections underwent antigen retrieval by boiling in sodium citrate solution for 20 min. Phospho-histone H3 (pH3) and Ki67 staining was followed by blocking in 10% goat serum for 20 min and incubation with primary antibodies against pH3 (1:100, rabbit monoclonal, Millipore, USA), Ki67 (1:100, rabbit polyclonal, Abcam, UK) or Aurora B (1:500, rabbit polyclonal, Abcam, UK) and cardiac troponin T (cTnT, 1:500, mouse monoclonal, Abcam, UK) overnight. Anti-rabbit and anti-mouse secondary antibodies conjugated to Alexa Fluor 488 or 594 were used for visualization by microscopy. Nuclei were visualized with 4', 6'-diamidino-phenylindole (DAPI).

For BrdU staining, DNA was then denatured by incubating slides in 1 N HCl for 10 min on ice, 2 N HCl for 10 min at room temperature, and 2 N HCl for 30 min at 37°C. HCl was neutralized by immersing slides twice in 0.1 M borate buffer, pH 8.5. The slides were then permeabilized with 0.5% Triton X-100 for 10 min. After the slides were blocked with 10% goat serum for 20 min, they were incubated with primary antibodies against BrdU (1:400, mouse monoclonal, Cell Signaling, USA) and cardiac troponin T (cTnT, 1:500, rabbit polyclonal, Abcam, UK) overnight. Anti-mouse and anti-rabbit secondary antibodies conjugated to Alexa Fluor 488 or 594 were then used.

EdU was detected with a Click-iT® EdU Alexa Fluor® 488 Imaging Kit (Invitrogen). Imaging was performed on a Leica DM6000B&DFC450C microscope or on a Leica SP8 confocal microscope. To quantify the percentage of Ki67-, pH3- and BrdU-positive cardiomyocytes, we analyzed five fields randomly captured from the third section of each heart to calculate the number of positively labeled and total cardiomyocytes, and then, the mean percentage of positive cardiomyocytes for each mouse was generated.

### Quantitative RT-PCR

The total RNA isolated using TRIzol reagent was quantified by ultraviolet (UV) spectrophotometry. cDNA was synthesized from 2 μg of total RNA using MMLV reverse transcriptase (Invitrogen). Real-time PCR was performed with SYBR Green detection. An ABI Prism 7300 sequence detection system (Applied Biosystems) was used for the PCR cycling reaction, real-time data collection, and analysis. GAPDH was selected as the reference gene. The relative transcript levels were quantified by the 2^-ΔΔCT^ method. The qRT-PCR primers are listed in Table 2.

### Western blot analysis

Samples from cultured cells were homogenized in lysis buffer with protease inhibitors, and total protein was extracted. Proteins were mixed with SDS sample buffer and loaded onto 4-12% gradient SDS-PAGE gels. The separated proteins were transferred onto nitrocellulose membranes by the Dry Blotting System (Invitrogen). The membranes were first probed with a specific primary antibody including pYAP (Ser127, Cell Signaling), YAP (Cell Signaling), pERK (Cell Signaling), ERK (Cell Signaling), pAKT (Cell Signaling), AKT (Cell Signaling), p-Smad1/5 (Cell Signaling), Smad1 (Cell Signaling), p-Smad2/3 (Cell Signaling) and GAPDH (Sigma) antibodies and then incubated with an appropriate secondary antibody, followed by visualization with ECL reagents (Thermo, USA).

### Statistics

All data are presented as the mean±SEM. We performed the homogeneity test of variance by GraphPad Prism 8, and all data passed the homogeneity test. For normally distributed quantitative data, Student's unpaired t-test was used to test statistical significance in two group comparisons. If the data were not normally distributed, a nonparametric test was performed. For analysis of data containing more than two groups, ANOVA with Tukey's pairwise post hoc test was used to compare means. All tests were performed using GraphPad Prism 8 software. A value of *P*<0.05 was considered statistically significant.

## Supplementary Material

Supplementary figures and tables.

## Figures and Tables

**Table 1:** List of primer sequences used for LPA_1_ and LPA_3_ knockout mice genotype identification

primer name	Sequence (5' to 3')
LPA_1_-A1KONew	ATCTGTGAAGCAAAGTCCTAAG
LPA_1_-Vzg.is2Fix	AGGAGTCTTGTGTTGCCTGTC
LPA_1_-A1IntRev	GATAGACTCATTGTAGAAGCAC
LPA_3_-A3e1b	TGACAAGCGCATGGACTTTTTC
LPA_3_-A3e1c	GAAGAAATCCGCAGCAGCTAA
LPA_3_-A3New F	GCACGAGACTAGTGAGACGTGCTAC

**Table 2:** List of primer sequences used for qRT-PCR

Primer name	Sequence (5' to 3')
LPA_3_ fwd	GTCTTAGGCGCCTTCGTGG
LPA_3_ rev	TTGCACGTTACACTGCTTGC
GAPDH fwd	TTGCACGTTACACTGCTTGC
GAPDH rev	GTGGTCATGAGCCCTTCCA

## Abbreviations

LPA: Lysophosphatidic acid
LPA_1/3_: Lysophosphatidic acid receptor 1/3
MI: myocardial infarction
WT: wild-type
KO: knockout
cTnT: cardiac troponin T
LVEF: left ventricular ejection fraction
LVFS: left ventricular fraction shortening
WGA: wheat germ agglutinin
TUNEL: terminal dexynucleotidyl transferase(TdT)-mediated dUTP nick end labeling
AAV9: Adeno-associated virus
CCK-8: cell counting kit-8
siRNA: small interfering RNA
RNA-seq: RNA sequencing
LAD: left anterior descending
RFP: red fluorescent protein
