# Supplementary material for: LPA3-mediated lysophosphatidic acid signaling promotes postnatal heart regeneration in mice
Source: Theranostics. 2020 Aug 29;10(24):10892–907. doi: 10.7150/thno.47913 (PMC7532668; doi:10.7150/thno.47913)

LPA3-mediated lysophosphatidic acid signaling promotes postnatal heart regeneration in mice

Fang Wang [1 #], Si Liu [2, 1 #], Jianqiu Pei [1#], Lin Cai [1], Ning Liu [4], Tian Liang [4], Xiaoxuan Dong [4], Xiangfeng Cong[1], Jerold Chun [3], Jinghai Chen [4 *], Shengshou Hu [1 *], Xi Chen [1 *]

Supplemental material:

Supplemental Figure 1. Cardiomyocyte apoptosis in the LPA_3 KO and wild-type control mice. (A) Quantification showing that no change in TUNEL signals was detected in the P4 and P7 hearts of the LPA_3 KO mice compared with the controls. (B) LPA_3 knockout did not influence the number of apoptotic cardiomyocytes after MI. n=5 mice in each group. Data are presented as the mean ± SEM; nonsignificant (N/S), $P > 0.05$.

Supplemental Figure 2. Validation of cardiac-specific LPA_3 expression induced by AAV9-cTNT-LPA_3-3Flag. (A) AAV9:LPA_3 or AAV9:EGFP viruses were injected subcutaneously into the P1 mice. Seven days or 21 days later, the hearts were analyzed by western blots, qRT-PCR and immunofluorescence. (B) Western blot analysis confirmed the expression of 3Flag-LPA_3 in the AAV9:LPA_3 group but not in the AAV9:EGFP group. (C) qRT-PCR confirmed that the expression of LPA_3 in the AAV:LPA_3 group increased 3-fold at P7 and 15-fold at P28 compared with that of

AAV9:EGFP. (D) AAV9:LPA_3 expressed Flag-tagged LPA_3 in the membrane of cardiomyocytes, scale bar =200 μm. Data are the mean ± SEM; AAV-EGFP, n = 3; AAV-LPA_3, n = 3; "n" indicates the number of mice.

Supplemental Figure 3. AAV9:LPA_3 has no effect on the healthy heart. (A). pH3 and Ki67 immunofluorescence of cardiomyocytes in the healthy hearts of the AAV9:LPA_3 and control adult mice at P8. Scale bar =50 μm. (B) Representative sections of hematoxylin-eosin staining and the HW/BW ratio at P8 and P7 of AAV9. Data are presented as the mean ± SEM; nonsignificant (N/S), $P > 0.05$; n=5 mice per group.

Supplemental Figure 4. Validation of the efficiency of siRNA. (A) LPA_3 was knocked down by siRNA and verified by qRT-PCR. (B) YAP was knocked down by si-YAP and verified by Western blot. n=3 samples of each group; **$P<0.01$; ***$P<0.001$.

Supplemental figure 1

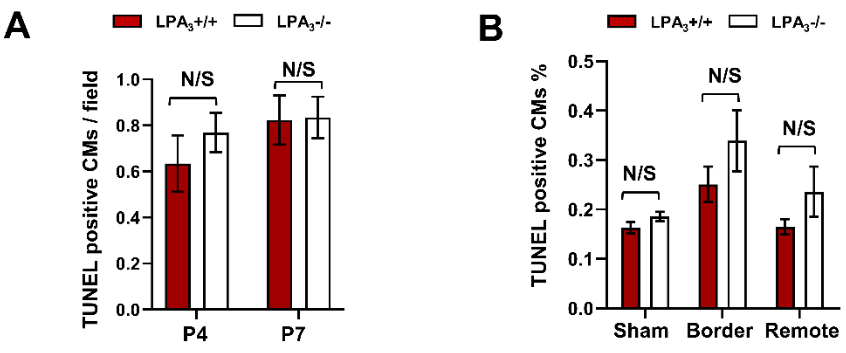

Supplemental figure 2

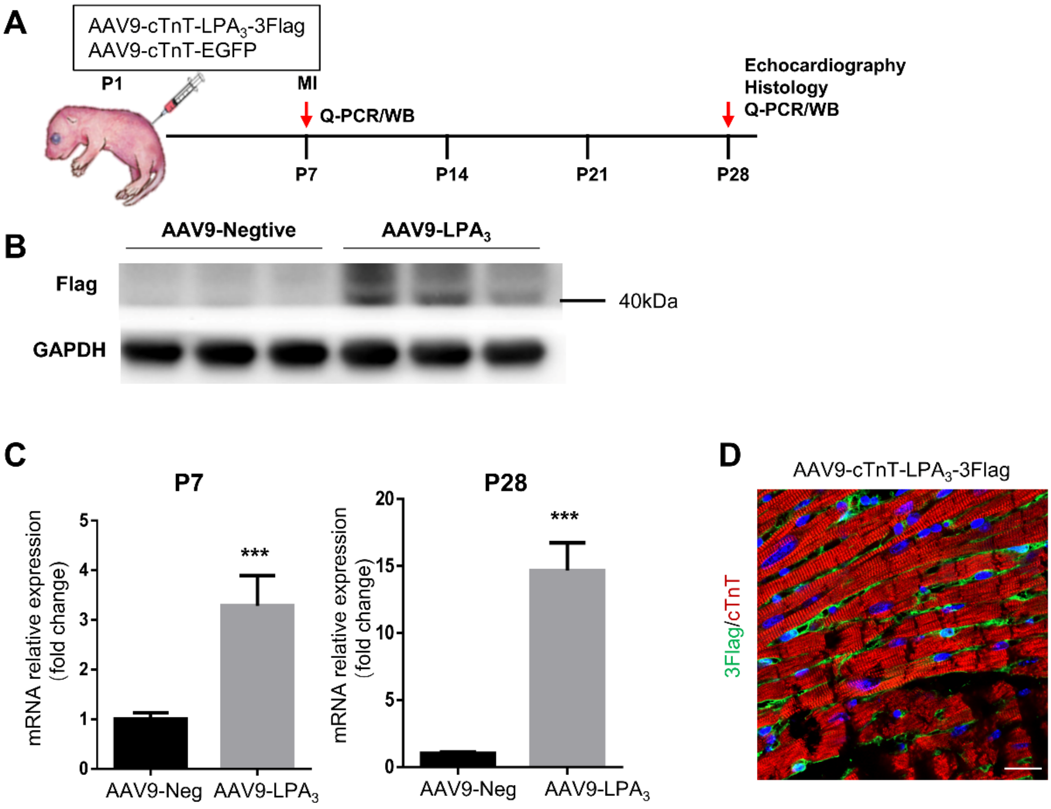

Supplemental figure 3

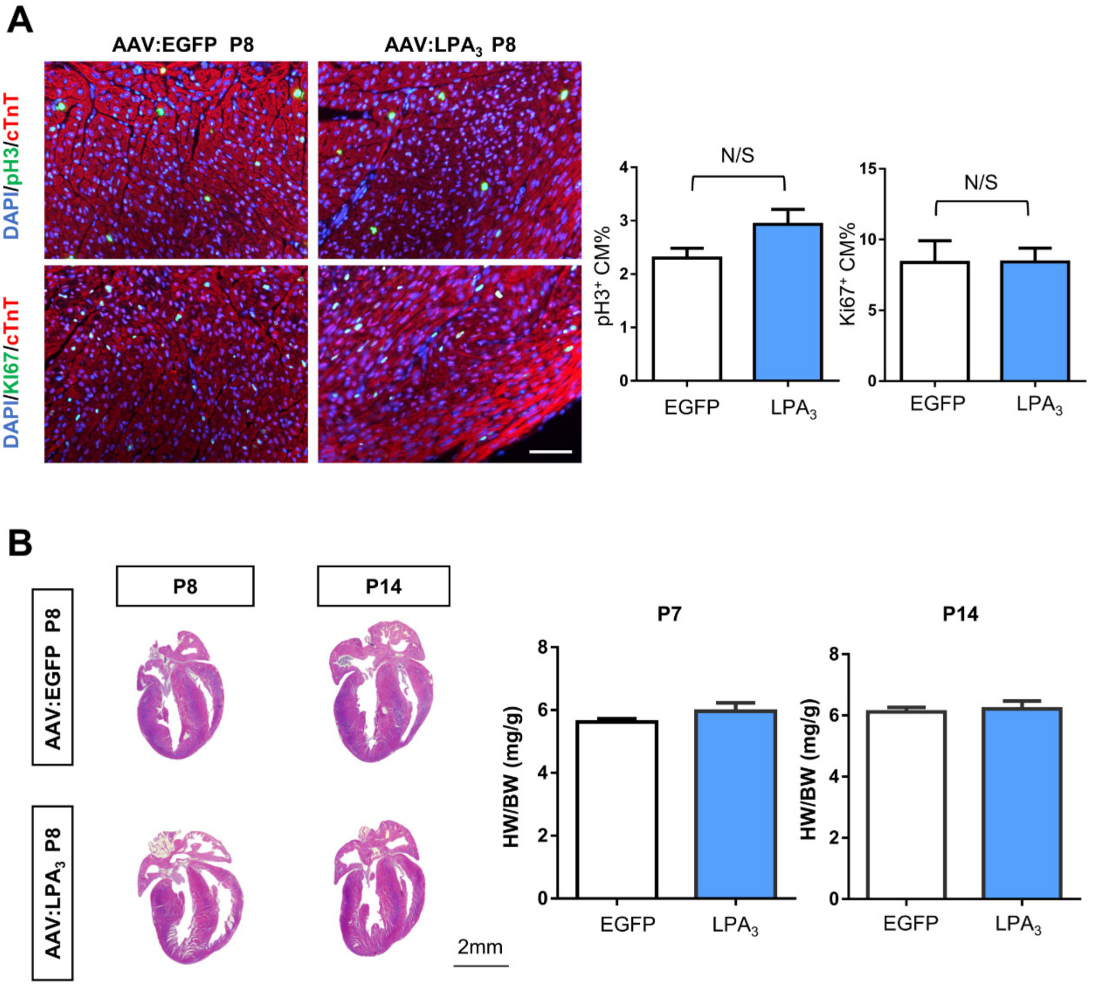

Supplemental figure 4

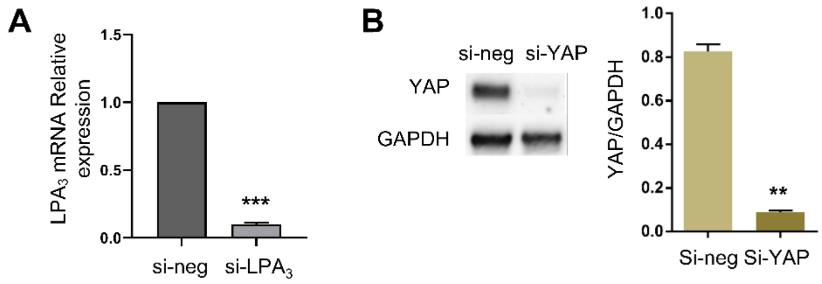

Supplement: Supplementary file 1 — Supplementary figures and tables. [file thnov10p10892s1.pdf]
